# “It’s Very Stressful for Children”: Elementary School-Aged Children’s Psychological Wellbeing during COVID-19 in Canada

**DOI:** 10.3390/children8121185

**Published:** 2021-12-15

**Authors:** Laena Maunula, Julia Dabravolskaj, Katerina Maximova, Shannon Sim, Noreen Willows, Amanda S. Newton, Paul J. Veugelers

**Affiliations:** 1 School of Public Health, University of Alberta, 3-50E University Terrace, 8303 112 Street NW, Edmonton, AB T6G 1K4, Canada; laena@ualberta.ca (L.M.); dabravol@ualberta.ca (J.D.); smsim@ualberta.ca (S.S.); paul.veugelers@ualberta.ca (P.J.V.); 2 MAP Centre for Urban Health Solutions, Li Ka Shing Knowledge Institute, St. Michael’s Hospital, 209 Victoria Street, Toronto, ON M5B 1T8, Canada; 3 Dalla Lana School of Public Health, University of Toronto, 155 College St Room 500, Toronto, ON M5T 3M7, Canada; 4 Department of Agricultural, Food & Nutritional Science, Faculty of Agricultural, Life & Environmental Sciences, University of Alberta, Edmonton, AB T6G 2P5, Canada; noreen.willows@ualberta.ca; 5 Department of Pediatrics, Faculty of Medicine & Dentistry, University of Alberta, Edmonton, AB T6G 1C9, Canada; an6@ualberta.ca

**Keywords:** qualitative research, psychological wellbeing, children and adolescents, COVID-19 pandemic

## Abstract

Emerging evidence suggests that the COVID-19 pandemic and associated public health measures, including lockdowns and school closures, have been negatively affecting school-aged children’s psychological wellbeing. To identify supports required to mitigate the negative impacts of the COVID-19 pandemic, we gathered in-depth information on school-aged children’s and parents’ lived experiences of COVID-19 and perceptions of its impact on psychological wellbeing in grade 4–6 students in Canada. In this qualitative study, we conducted telephone-based semi-structured interviews with parents (*n* = 15) and their children (*n* = 16) from six schools in small and mid-sized northern prairie communities in Canada. Interviews were analyzed through thematic analysis. Three interrelated themes have emerged. First, the start of COVID-19 brought sudden and stressful changes to children’s lives. Second, disruptions to daily life led to feelings of boredom and lack of purpose. Third, limited opportunities for social interaction led to loneliness and an increase in screen time to seek social connection with peers. Results underscore the need for resilience building and the promotion of positive coping strategies to help school-aged children thrive in the event of future health crises or natural disasters.

## 1. Introduction

The COVID-19 pandemic resulted in the implementation of public health measures (school closures, cancellations of extracurricular activities, physical distancing, ‘stay home’ orders) that created a lot of hardship for large populations of school-aged children [1]. Severe limits on social interaction during childhood can lead to loneliness and social isolation [2,3]—major risk factors for future mental health problems [4]. Emerging research reports on declines in health-related quality of life and increases in psychopathology (e.g., depression, anxiety [5,6,7,8], suicidal ideation, and suicide attempts [9]) in school-aged children since the start of COVID-19. However, there is also a need to understand the contextual information concerning school-aged children’s lived experiences of COVID-19 and its impacts on their psychological wellbeing [10,11,12]. To date, only two qualitative studies, from Ireland [13] and India [14], have contributed such accounts. Given the varying intensity and duration of COVID-19 public health measures across countries and regions [15], our focus in this study is on the lived experiences and perceptions of the impact of the COVID-19 pandemic on psychological wellbeing among elementary school-aged children in Canada.

## 2. Materials and Methods

This research is part of a multi-method study (i.e., one using complementary methodologies to answer a common overall research goal [16]) that examined the impacts of COVID-19 on elementary school children in 20 APPLE (A Project Promoting healthy Living for Everyone) Schools in Western Canada. APPLE Schools is an innovative, internationally recognized, not-for-profit health promotion program targeting schools located in socioeconomically disadvantaged communities [17]. Following a Comprehensive School Health (CSH) approach [18], the program promotes healthy lifestyle behaviours and psychological wellbeing. The CSH approach, grounded in a socio-ecological model [19] of health, emphasizes wellbeing as essential for student achievement. The CSH approach shaped the development of the project objectives, interview guides, and data collection and was used to contextualize the findings. This research is situated within a post-positivist paradigm: realist in ontology, instrumental in epistemology [20].

To explore children’s and their parents’ perspectives simultaneously, we interviewed 15 parent–child pairs (i.e., 15 parents and 16 children) from six small and mid-sized communities (population < 15,000) in the provinces of Alberta and Manitoba, Canada, between December 2020 and February 2021. One child attended school in an urban area but lived on an acreage outside of the city. At the time of study participation, children were attending grades 4–6 and had returned to in-person learning. In Alberta and Manitoba, Canada, all public schools were closed from mid-March 2020 to the end of the school year in June 2020 [21]. Remote learning for students, either via videoconference or paper-based curriculum, continued through the end of the school year. Classrooms reopened in September 2020 but were suspended again for two weeks in January 2021. As part of fluctuating provincial restrictions on public gatherings and lockdowns, non-essential businesses and cultural venues were also closed, and extracurricular programmes for children were either suspended or operated in an online format, leaving school-aged children with few to no opportunities for social interaction or recreation. Nonetheless, APPLE Schools continued the active delivery of their programming during COVID-19 (including school closures and in-person learning), with a particular focus on maintaining school-aged children’s psychological wellbeing through mindfulness techniques and other strategies [22].

We used purposive, snowball, and convenience sampling techniques to recruit participants through a larger research study, whereby parents also completed a survey on their children’s psychological wellbeing and lifestyle behaviours. Every 15th survey respondent was contacted by email regarding participation in this study. Recruitment continued concurrently with survey administration until data saturation was reached. Each participant received a 30 CAD gift card to a local store of their choice.

Children and their parents were interviewed separately. The development of the child and parent interview guides was informed by our central research question—i.e., “What are the lived experiences and perceptions of the impact of the COVID-19 pandemic on psychological wellbeing of school-aged children?” Therefore, the child interview guide centred on children’s experience of COVID-19 broadly, with questions focusing on psychological wellbeing (e.g., emotions and feelings since the start of the pandemic and particularly during the lockdown, APPLE Schools supports), as well as lifestyle behaviours and experiences of remote learning. Similarly, the parent interview guide centred on parents’ perspectives of their child’s experiences with COVID-19, with questions focusing on children’s psychological wellbeing, as well as lifestyle behaviours, and parents’ perceptions of APPLE Schools support during COVID-19. Parent interviews lasted approximately an hour, and child interviews about 30 min. Demographic information was collected prior to interviews. Due to unreliable internet service in some communities, interviews were conducted by telephone rather than videoconferencing platforms. A qualitatively trained interviewer with a background in public perceptions of pandemics (LM) [23,24,25] conducted all interviews, taking particular care and time to build rapport and establish trust with participants [26]. The interviews were audio-recorded and transcribed verbatim.

The Health Research Ethics Board of the University of Alberta (Pro00061528, 30 November 2020) and participating school boards approved all study procedures. Each parent provided written consent to participate in the study and for the involvement of their child(ren). Assent was obtained from children prior to being interviewed.

Qualitative data emerged in response to the interviewer’s questions, informed by our research question, and were then thematically analyzed following the Braun and Clarke [27] approach. The thematic analysis consisted of five broad, looping, and recursive steps, beginning with close, layered reads and note-taking to become familiar with the data. Participant profiles were written to provide a high-level summary of each interview and serve as a counterpoint to the more granular coding of thematic analysis. Next, initial small units of meaning were organized into meaningful groups, thereby generating a set of initial codes. Inductive and primarily “data driven” coding of each transcript was performed manually [27] and resulted in numerous initial codes. These codes were then collapsed into larger descriptive themes, first within each transcript text, then across each transcript in the sample. As child and parent participants lacked commonality of experiences, the child and parent interview data were analyzed separately, with unique themes developed for the two datasets. The nascent themes were then reviewed and revised through discussions with the research team and through reading and reflecting on CSH to sensitize the analyst to concepts relevant to this framework. As a final step, descriptive themes were compared across parent and child datasets and refined to create a set of themes that spanned across both samples. These themes were further refined through the analytic act of producing the written analysis. Trustworthiness was ensured through peer debriefing sessions with the whole team, reflexive journaling during data collection and analysis, and keeping field notes for each interview and the research process [28] (pp. 163–194). This study follows the Standards for Reporting Qualitative Research [29].

## 3. Results

On average, children were 11 years old, and parents were 46 years old (Table 1). Three interrelated themes emerged from thematic analysis: (1) “It is very stressful for children”: Facing the onset of the pandemic; (2) “There was not a whole lot of purpose”: Navigating the new normal; and (3) “I literally want to jump through the window and run to school”: Struggling with a lack of social interaction.

### 3.1. “It Is Very Stressful for Children”: Facing the Onset of a Pandemic

Children and parents both expressed that the COVID-19 onset brought a myriad of sudden changes to which children rapidly had to adapt. Children described difficulties grappling with the lack of certainty and unfamiliar circumstances (e.g., physical distancing, mask-wearing, and staying home).

Child, 14b: I feel like it’s difficult. Cause this [has] never happened for our age and […] this is so new to us.

Child, 7b: I wish that they [grownups] knew that it [pandemic] is stressful … Very stressful for us children.

Parents noted the sudden changes and upheavals to daily life from public health restrictions: “I think it’s kind of changed him [child] in a sense. Which I think is changing children in general, because now they’re all worried about things that we didn’t have to worry about two years ago” (Parent, 9a). Some children were not concerned about getting infected due to a perceived low risk of severe outcomes: “I didn’t have anxiety about COVID. I know if I catch it I’m not going to die” (Child, 3b). However, others expressed fear of either becoming infected or infecting loved ones: “I’ve never had to wear a mask. I’ve never had to worry about people I love getting sick and dying” (Child, 8b). The children’s fear of infection, both their own and their loved ones, was also expressed by several parents:

Parent, 7a: My son has developed extreme anxiety to the outside world. He’s not even comfortable going into a grocery store. Like he has sheltered himself right off.

Parent 10a: But then on the drive home [child] scolded my husband … He got so upset with us. He cried and was very angry. So much so that we had to get my mom on the phone […] to talk to him and reassure him that it was okay … That was a very hard one for him. ‘Cause grandma’s very important to him.

Additional stressors arose from remote learning and ensuing changes in family dynamics, including increases in sibling fighting and tensions with parents concerning remote learning expectations. Unpredictability concerning how long the public health restrictions would last was a related concern for many children: “Kind of scary … Because we didn’t know how bad it would be getting” (Child, 11b); “I don’t know how I feel. Like I have bad anxiety and I feel like this is going to last forever” (Child 9b). Several parents noted improvements in children’s stress from practicing mindfulness activities at home, such as deep breathing exercises, or through having personalized check-ins with teachers and APPLE Schools staff:

Parent, 5a: […] [M]indfulness was definitely a coping strategy and something that helped … identify maybe some feelings that you were having about changes.

Parent, 15a: When they [teachers] connected with the kids person-to-person whether it was a phone call, a virtual meet, or like a drive by and see your teacher. […] [T]hat was way more impactful than the worksheet or the email or the written word.

### 3.2. “There Was Not a Whole Lot of Purpose”: Navigating the New Normal

A second theme concerned the impact of changes in daily schedules and routines resulting from the COVID-19 public health restrictions. Children described experiencing boredom due to the lack of available activities: “I just, I hate being bored. I love being more active. But since [COVID-19 started], everything’s really just changed” (Child, 14b). For children who experienced delays when switching from in-person to remote learning, the monotony was exacerbated: “It was the same thing all day. And then online school for us only came about five weeks in. So, for those five weeks there wasn’t a whole lot of purpose” (Child, 9b). Parents similarly indicated that lockdowns and the sudden reduction in regular, structured activities also exacerbated monotony and hence made for difficult adjustments: “My youngest one is very active … And all of a sudden it was, ‘You’re at home and that’s it’. So mentally she did not adjust. She still hasn’t adjusted” (Parent, 7a).

Children commonly described increased screen time, including playing video games and watching movies, to combat boredom:

Child, 2b: Kind of feels like you want to just … fall asleep or just watch shows, because you have nothing to do.

Child, 14 b: […] I’ve been spending more time on my phone. … [N]ow people are getting bored … and so, all they do is just sit on their phones.

Several children identified increased screen time as their response to being “stuck” in the house: “I think I did a lot more of [screen time] during lockdown. Because usually [before COVID-19] I’d be like, out and about all day. But now I was just stuck at home and locked away” (Child, 2b).

Parents similarly acknowledged screen time among children had increased as a means of coping with boredom and a lack of other options for downtime or relaxation and to provide some space for each other. Yet, parents expressed frustration with the amount of screen time and how this may reflect on their parenting abilities:

Parent 4a: Well screen time’s not good, but […] we’re using it as a crutch, that’s for sure. Just because we have no space from each other too. Or no [extra]curricular. Like there’s nothing extra for any of us.

Parent 12a: I worry that I let them on there [screens] way too much … I feel kind of like a bad parent … But I just feel like, you know what? If they want to play that game, I find myself just letting them do that. Because what else are they supposed to do, right?

Other than increased screen time, to cope with the boredom, one child described an ad hoc daily bicycle riding group they created with neighbourhood children. The child identified participating in daily physical activity with peers during lockdown (while adhering to physical distancing guidelines) as being helpful:

Child, 9b: Well, I first started when I called my friend and said, “Hey, do you want to go for a bike ride? I’m tired of it”. And he said, “Sure”. And we went down to the lake and sat down on the dock. It was cool. And then we started asking more people … And we’d go around the block, or to the stop sign, which is like three kilometers. So … it got rid of the boredom cause there’s always something to see.

According to several parents and children, caring for pets and animals helped children cope with the monotony: “We decided to foster cats. Which has been a huge, I think morale booster for the kids. So, a pleasant distraction” (Parent 8a); “Playing with the goats, playing with the dogs … We get to help with the goat chores” (Child, 5b).

When asked if anything helped improve their moods during remote learning, some children mentioned virtual or socially distanced school challenges and special events.

Child, 15b: We had a beach day and we brought all our pool noodles and stuff and drove to school. And all our teachers were out there waving to us, with their beach themed stuff … It was fun to actually see all our teachers.

### 3.3. “I Literally Want to Jump through the Window and Run to School”: Struggling with a Lack of Social Interaction

A theme expressed by every child was sadness and frustration regarding the reduced amount and quality of social interaction with friends during COVID-19, particularly during remote learning and lockdowns. Loneliness or missing friends was often among the first things children mentioned when asked to describe their experience of COVID-19: “I’m used to seeing my friends a lot, and then that didn’t happen for a long time. And it was kind of dark for a little bit” (Child, 9b). Several children identified the lack of social interaction as the most significant impact of COVID-19. One child recalled they “didn’t laugh for three months in that quarantine period” due to lack of “human interaction” (Child, 9b). The following quotation exemplifies the poignancy of missing friends: “[N]ot being able to see them [friends] and go to their houses right now, it’s very—it just doesn’t feel good … [It] makes me kind of sad and I am sure it makes my friends sad” (Child, 10b).

Parent participants echoed the sentiment of children’s loneliness, particularly during remote learning and lockdowns.

Parent, 11a: [Child] wasn’t normally a kid that you [would] say, ‘He loves school’ … Or like, he would be trying to get out of school […] But then after that break, we had to self-isolate for a week. And he cried everyday to go to school. He did not want to miss it … They’re very social, they want to be with their friends. They want to see each other.

Many children and parents recounted that a return to in-person learning, and subsequently, increased interaction with friends, led to elevated moods: “I felt really happy because we get to see everyone again” (Child, 13b); “They were very happy to be back in school, able to see their friends” (Parent, 3a).

Limited socializing prompted children to seek opportunities to interact with friends virtually, through video game chat functions and video calls. Several children and parents noted that children began using technology for the first time to socialize during COVID-19.

Child, 10b: The thing that brought me down a lot … would probably be because I couldn’t see my friends and stuff. I would have to video call them or something.

Child, 13b: I started [to talk with friends on Zoom or Google Meet] when COVID was starting and I didn’t get to see people as much.

According to both parents and children, connecting virtually with friends tended to alleviate sadness:

Parent, 3a: At times they were depressed, like, ‘I want to see my friends.’ But then they would Facetime with their friends or play an online game and that would help. It’s amazing how much social interaction can brighten a child’s mood.

Child, 7b: Like sometimes I feel so anxious about wanting to see my friends, I literally want to jump through the window and run to school and see them. But luckily, I just communicate with them on Snapchat and stuff.

Although several parents noted that virtual social interaction was not ideal and lacked the nuance and depth of face-to-face socializing (“It’s just an awkward social way to interact” (Parent, 8a)), increased screen time was something many parents reluctantly allowed to facilitate children’s social needs.

Parent, 2a: At first, I was like, ‘We got to limit that screen time.’ And I got to admit, throughout the process, it did increase because that was her avenue to still have social communication with friends. And honestly, when it was cold and yucky at times too, just to not [be] staring at a wall. Because what’s the worst of the evils here, right?

Several parents expressed concern over the increase in screen time and its impact on children’s psychological wellbeing: “[…] [I]t’s almost become like an addiction, which I’ve worked very hard previous to this, to avoid. And it’s not something I ever wanted for my kids” (Parent, 6a).

## 4. Discussion

We explored the lived experiences of COVID-19 in elementary school-aged children and their parents, focusing on the impacts of COVID-19 on children’s psychological wellbeing. Three interrelated themes have emerged in the thematic analysis of qualitative data gleaned from children and parents’ interviews. First, the start of COVID-19 brought sudden and stressful changes to children’s lives. Second, disruptions to daily life led to feelings of boredom and lack of purpose. Third, limited opportunities for social interaction led to loneliness and increased screen time to seek social connection with peers. Below, we discuss these findings in more detail and provide recommendations at the individual, family, community, and school levels informed by these results.

Participants described children feeling boredom due to monotony, loneliness due to social isolation, and worrying due to uncertainty imposed by sudden pandemic-related changes. These results corroborate a recent systematic review of 15 observational studies examining the effects of COVID-19 on children’s psychological wellbeing [30]. Children increased screen time to cope with boredom and limited options for recreation and social interaction during COVID-19 lockdowns. These findings reinforce the need to foster resilience [31], which is pivotal for the maintenance and promotion of psychological wellbeing among children [32], and helps buffer negative psychosocial outcomes [33] and manage boredom [34,35,36].

Promoting resilience at the family, community, and school levels will help support children to cope with future pandemics or natural disasters. Since children expressed difficulty grappling with boredom and monotony during lockdowns, strategies to build children’s resilience at the family level may include nurturing creativity through art-making and activities that allow for the expression of strong emotions [37,38], bolstering children’s self-esteem and self-worth [39], providing unstructured time with enriching activities, spending more time together as a family [40,41], addressing family conflicts in a timely and compassionate manner, and providing opportunities for children to talk about COVID-19 as a way to process it [42]. To promote community resilience, health promotion programming targeting community connectedness [43], such as peer support networks or “buddy systems” [44], should be co-designed with community stakeholders. To buffer negative mental health risks over time, promoting resilience in schools can foster positive coping skills, such as help-seeking, positive appraisal and thinking, and problem-solving [45]. During COVID-19, APPLE Schools programming focused on resilience-building strategies to maintain children’s psychological wellbeing [22], which is in line with strategies outlined in a recent School for Health in Europe Network Foundation guideline that focuses on implementation strategies based on the CSH (or health-promoting schools) approach during the COVID-19 pandemic [46].

On the individual (student) level, healthy lifestyle behaviours (exercising, spending time outdoors [47], eating well, good quality sleep [48]) and emotion-focused coping strategies (relaxation techniques [49], mindfulness techniques, and philosophical and moral reflections [50]) have been associated with better psychological wellbeing in children during COVID-19. In our findings, children who engaged in regular physical activity (e.g., socially distanced bicycle riding with friends) or practiced mindfulness, found those activities to be helpful in coping with stress, boredom, and loneliness.

Given the importance of friendships as a crucial source of attachment, intimacy, and social support [51], loneliness and missing social interaction was a major stressor for children. While adolescents with low social support appear to be at a higher risk of depression and anxiety [52], a study of American youth during COVID-19 reported a decrease in technology-facilitated interactions with friends, a lack of emotional connection, and a decrease in friend support [40]. Although several children in our study also claimed that screen interaction with friends was not as nuanced as time spent face-to-face, online interaction is the safest way to receive emotional and informational support in the context of the ongoing pandemic [53,54]. Ensuring that families have access to the Internet and technology through schools and/or community organizations [55] and advocating for children to use technology safely to socialize with peers (playing online games together, chatting, celebrating milestones and special occasions) [39] can alleviate loneliness and maintain social skills.

This study has several strengths. We interviewed parent–child pairs, providing a unique opportunity to explore how both parents and children perceived children’s psychological wellbeing. The timing was another strength, as we collected data when the COVID-19 experience was current. Several limitations warrant consideration. Participants were mainly from families with relatively high household education and/or income, and therefore, possibly more articulate, health-conscious, and active in children’s schooling. Perspectives of those who may not readily volunteer for health studies are needed; however, this limitation is common in qualitative research. Interviews were conducted by telephone, thus making information typically communicated by body language and facial expressions unavailable. To mitigate this limitation, the interviewer paid close attention to non-verbal details, such as pauses, tone changes, sighs, and laughter. These non-verbal cues formed part of the interview interaction, helping shape its flow by following the participants’ lead [56]. Lastly, during some interviews, parents remained close by while children were being interviewed. This might have affected children’s willingness or readiness to share negative experiences.

## 5. Conclusions

COVID-19 public health measures, including long periods of lockdowns, school closures and remote learning, have upheaved children’s daily lives and routines, resulting in feelings of boredom, lack of purpose, and loneliness. Fostering resilience through school- and community-based strategies can help school-aged children cope with future pandemics or natural disasters and thrive despite challenging life circumstances. More research exploring the existing theoretical frameworks on resilience and coping frameworks in the context of COVID-19 is warranted.

## Figures and Tables

**Table 1:** Characteristics of Children (*n* = 16) and Parents (*n* = 15).

Characteristic	Mean (Range) or *n* (%)
Children	
Age in years	11.2 (9–12)
Born in Canada	16 (100%)
Province of residence	
Alberta	10 (62.5%)
Manitoba	6 (37.5%)
Have siblings	15 (93.7%)
**Parents**	
Age in years	46 (31–50)
Born in Canada	15 (100%)
English as the language in household	15 (100%)
Number of children	2.6 (1–4)
Highest level of education	
Secondary (7–12)	1 (6.6%)
Community/Technical College	7 (46.8%)
University	6 (40%)
Graduate University	1 (6.6%)
Current marital status	
Common-law	1 (6.6%)
Married (and not separated)	12 (80%)
Divorced	2 (13.3%)
Employment has changed since the start of COVID-19	2 (13.3%)
Current household income (CAD)	
25,001–50,000	1 (6.7%)
50,001–75,000	2 (13.3%)
75,001–100,000	5 (33.4%)
More than 100,000	7 (46.6%)
Region of residence	
Rural	3 (20.0%)
Small PC	12 (80.0%)

PC: population centre.

## Data Availability

The data are not publicly available due to confidentiality concerns.
